# Outcomes of neonatal hypothermia among very low birth weight infants: a Meta-analysis

**DOI:** 10.1186/s40748-021-00134-6

**Published:** 2021-09-15

**Authors:** Sagad Omer Obeid Mohamed, Sara Mohamed Ibrahim Ahmed, Reem Jamal Yousif Khidir, Mutaz Tarig Hassan Ahmed Shaheen, Mosab Hussen Mostafa Adam, Basil Abubakr Yagoub Ibrahim, Esra Osama Abdelrahman Elmahdi, Abubaker Shadoul Mohamed Farah

**Affiliations:** Faculty of Medicine, University of Khartoum, Alqasr Avenue, P.O. Box 102, Khartoum, Sudan

**Keywords:** Hypothermia, Very low birth weight, Meta-analysis

## Abstract

**Background:**

Neonatal admission hypothermia (HT) is a frequently encountered problem in neonatal intensive care units (NICUs) and it has been linked to a higher risk of mortality and morbidity. However, there is a disparity in data in the existing literature regarding the prevalence and outcomes associated with HT in very low birth weight (VLBW) infants. This review aimed to provide further summary and analyses of the association between HT and adverse clinical outcomes in VLBW infants.

**Methods:**

In July 2020, we conducted this review according to the Preferred Reporting Items for Systematic Reviews and Meta-Analyses guidelines. A systematic database search was conducted in MEDLINE (PubMed), Google Scholar, ScienceDirect, World Health Organization Virtual Health Library, Cochrane Library databases, and System for Information on Grey Literature in Europe (SIGLE). We included studies that assessed the prevalence of HT and/or the association between HT and any adverse outcomes in VLBW infants. We calculated the pooled prevalence and Odds Ratio (OR) estimates with the corresponding 95% Confidence Interval (CI) using the Comprehensive meta-analysis software version 3.3 (Biostat, Engle-wood, NJ, USA; http://www.Meta-Analysis.com).

**Results:**

Eighteen studies that fulfilled the eligibility criteria were meta-analyzed. The pooled prevalence of HT among VLBW infants was 48.3% (95% CI, 42.0–54.7%). HT in VLBW infants was significantly associated with mortality (OR = 1.89; 1.72–2.09), intra-ventricular hemorrhage (OR = 1.86; 1.09–3.14), bronchopulmonary dysplasia (OR = 1.28; 1.16–1.40), neonatal sepsis (OR = 1.47; 1.09–2.49), and retinopathy of prematurity (OR = 1.45; 1.28–1.72).

**Conclusion:**

Neonatal HT rate is high in VLBW infants and it is a risk factor for mortality and morbidity in VLBW infants. This review provides a comprehensive view of the prevalence and outcomes of HT in VLBW infants.

## Background

Neonatal deaths account for 40% of all under-five-years mortality and most of the deaths occur in developing countries [1, 2]. In particular, low birth weight accounts for 60–80% of all neonatal deaths [2]. Causes of neonatal mortality include complications of prematurity, infections, birth asphyxia, congenital anomalies, and others [3, 4].

Several studies have reported an association between neonatal admission hypothermia (HT) and increased risk of mortality and morbidity in VLBW infants [5–13]. These outcomes associated with HT are particularly evident in VLBW newborns due to their impaired and easily overwhelmed temperature regulatory mechanism, which is mainly due to high surface area to mass ratio, decreased subcutaneous fat, lack of shivering response, and inadequate brown fat [2, 10–12]. Laptook et al. found in an adjusted analysis that with each 1 °C decrease in body temperature of VLBW infants, sepsis and mortality risks increase by 11 and 28%, respectively [13].

According to the World Health Organization (WHO), HT is defined as a core body temperature of less than 36.5 °C and the severity is sub-classified into three grades: Cold stress or mild HT (36.0–36.4 °C), moderate HT (32.0–35.9 °C), and severe HT (less than 32 °C) [14]. For each of these classifications, there are guidelines in place for responding to or managing HT [15]. Risk factors for HT include low gestational age, asphyxia, improper control of the thermal environment, inadequate breastfeeding, mode and place of delivery [16–19]. In addition, De Almeida et al. showed that maternal HT prior to delivery nearly doubles the chance that a newly born infant will have HT at five minutes after birth, indicating that maternal thermal care is an important measure for the prevention of neonatal HT [20].

Management of HT is considered a cornerstone for neonatal resuscitation as it reduces rates of neonatal mortality and morbidity [2, 21, 22]. According to the United Nations Children’s Fund (UNICEF), these interventions can reduce the outcomes of HT by 18–42% [21]. These interventions that have been proposed to prevent HT in newborn infants include using a plastic cap, polyethylene bag wrapping, thermal mattress, controlling the room temperature, and active warming during cesarean delivery [9, 17, 23, 24]. A quality improvement project on the implementation of thermoregulatory interventions showed a significant reduction of neonatal HT, which in turn improved neonatal outcomes [25].

There is disparity in reported data on the prevalence of HT in VLBW infants and the associated outcomes with HT despite the high burden of VLBW infant deliveries and HT [1, 11]. Therefore, we conducted this review to assess the burden and adverse clinical outcomes associated with HT among VLBW infants.

## Methods

### Search strategy and inclusion criteria

The methodology was developed from the Preferred Reporting Items for Systematic Reviews and Meta-Analyses (PRISMA) statement [26]. We performed a systematic literature search using the electronic databases of MEDLINE (PubMed), Google Scholar, ScienceDirect, WHO Virtual health library, Cochrane Library databases, and System for Information on Grey Literature in Europe (SIGLE) without limitations regarding sex, race, geographical area, or publication date. The search terms used were “low birth weight”, “very low birth weight”, “LBW”, “VLBW”, “hypothermia”, and “low body temperature”. Also, we reviewed the articles referenced by those identified articles in this search to ensure no possible relevant articles were missed.

The retrieved publications were imported into Rayyan software (QCRI, Doha, Qatar; http://rayyan.qcri.org) to expedite initial titles/abstracts screening and duplicates deletion [27]. The target population was the VLBW infants (those who were < 1500 g) and the criteria for articles inclusion were cross-sectional, case-control, or cohort studies that assessed the prevalence of HT and/or association between HT and any adverse outcome in VLBW infants. We excluded case reports, editorials, reviews, abstracts, non-English articles, and studies without sufficient data of interest. The titles and abstracts of all articles retrieved from this search were screened for potential inclusion in this review. Then, the full texts of studies deemed to be relevant were reviewed for inclusion according to the defined eligibility criteria.

### Quality assessment and data extraction

Quality assessment of the included studies was done using the Joanna Briggs Institute critical appraisal checklists, which were set to assess the methodological quality of studies and to determine to what extent a study addressed the possibility of bias in study design, conduct, and data analysis [28]. Four independent reviewers extracted the relevant information using a designed data extraction form. Any disparity among the reviewers was resolved by discussion and consensus. For qualitative and quantitative data syntheses, we extracted the following information from each article and recorded them in a Microsoft Excel spreadsheet: authors, year of publication, study region, outcomes measured in the study, reference level used for definition of HT, number of VLBW infants, infants with HT, and infants with and without an adverse outcome in both hypothermic and non-hypothermic groups.

### Statistical analysis

The statistical analyses were carried out by using Comprehensive meta-analysis software version 3.3 (Biostat, Engle-wood, NJ, USA; http://www.Meta-Analysis.com). The heterogeneity was assessed through I^2^ test, which describes the percentage of variability in the effect estimates. If high heterogeneity was detected, we calculated the pooled summary prevalence and OR from the random-effects models. We conducted a meta-regression analysis to determine the extent to which the continuous variables moderated the overall results. Publication bias was determined through Begg’s test, Egger’s test, and visual examination of the funnel plot [29, 30]. If a publication bias was found, the Duvall and Tweedie’s trim and fill method was used to add possible missing studies and to calculate the adjusted pooled value [31].

## Results

### Studies characteristics and prevalence of HT among VLBW infants

**Fig. 1:**
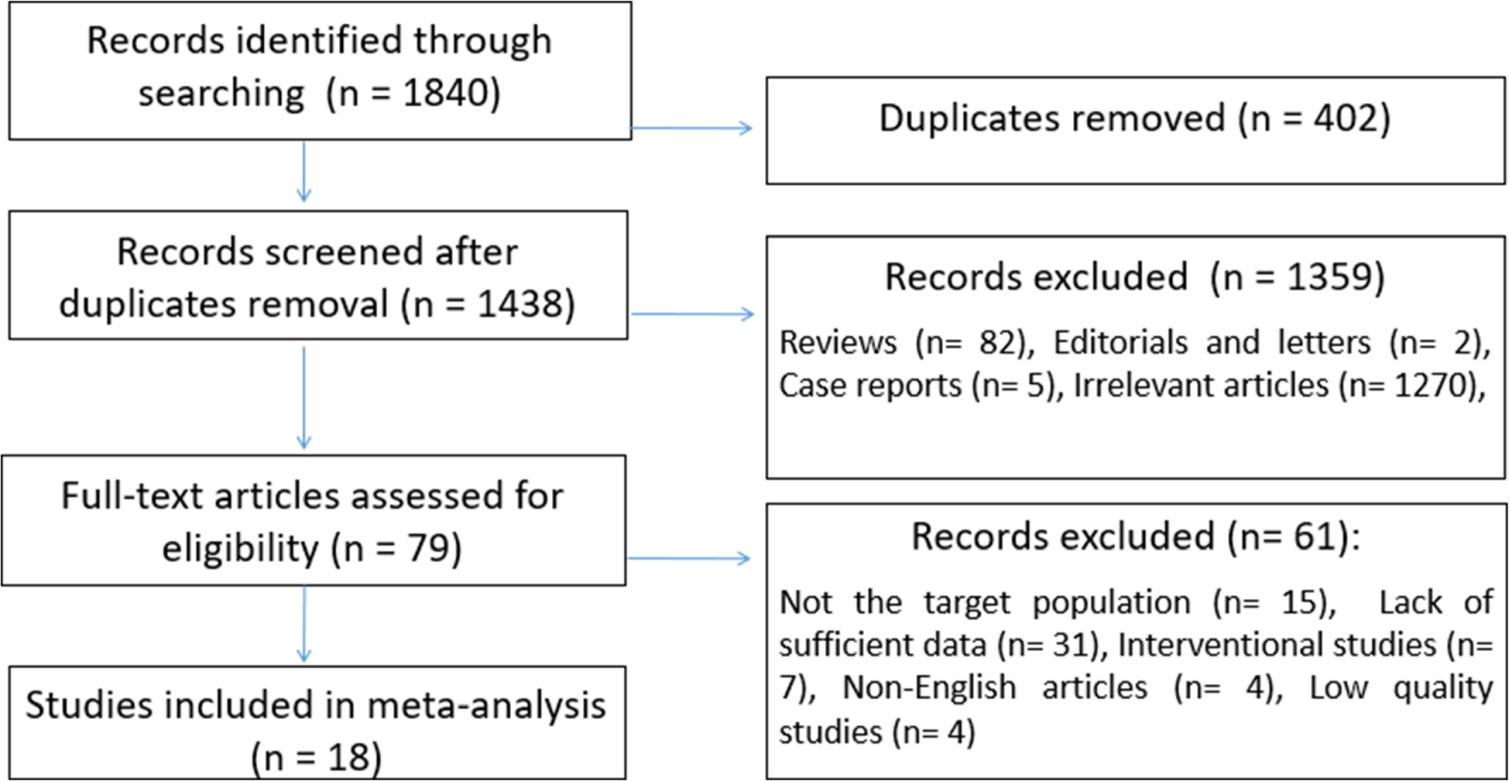
Flow chart for study selection process

Our search retrieved records for 1840 published articles and the records screened after the removal of duplicates were 1438 articles. We excluded 1359 records in the title/abstracts screening phase after applying the exclusion criteria. Then, full texts of 79 studies were assessed for eligibility and quality. Lastly, a total of 18 studies conducted from 1981 to 2020 were included in the meta-analysis [5–7, 9–13, 20, 24, 32–39]. The studies selection process and the main findings are presented in (Fig. 1) and (Table. 1).

**Table 1:** Descriptive summary of the studies included in the review

Study	Study Year	Country	Adverse outcomes found to be associated with HT	HT cut point (°C)	No. of VLBW infants	No. (%) of infants with HT
Levi et al. [38]	1981	USA	Mortality	36.0	71	53 (74.6%)
Neonatal Data Collection Group [33]	1993	Malaysia	Mortality	36.0	824	280 (33.9%)
Malaysian VLBW group [34]	1997	Malaysia	Mortality	36.0	868	288 (33.2%)
Laptook et al. [13]	2003	USA	Mortality (28% increase per 1 °C decrease) and sepsis (11% increase per 1 °C decrease)	36.0	5277	2475 (46.9%)
Sodemann et al. [39]	2003	Guinea-Bissau	Mortality	34.5	23	13 (56.5%)
Demirel et al. [35]	2006	Turkey	BPD	36.0	106	32 (30.1%)
Miller et al. [6]	2007	USA	Mortality, IVH, and sepsis	36.5	8782	4953 (56.4%)
Ogunlesi et al. [32]	2008	Nigeria	Mortality	36.5	15	14 (93.3%)
Ting et al. [7]	2011	Canada	Mortality, sepsis and adverse neurodevelopmental outcomes	36.5	2739	968 (35.3%)
Audeh et al. [24]	2011	Israel	Non	35.5	271	100 (36.9%)
Lyu et al. [37]	2012	Canada	Mortality, severe neurological injury, severe ROP, NEC, and BPD	36.5	9833	3516 (35.7%)
De Almeida et al. [20]	2012	Brazil	Mortality	36.0	1764	900 (51.0%)
Guinsburg et al. [12]	2015	Brazil	Unfavorable outcome (in-hospital mortality or survival with BPD, IVH III/IV, PVL and/or ROP)	36.0	2646	868 (32.8%)
Chang et al. [10]	2015	Taiwan	Mortality and RDS	36.5	341	262 (76.8%)
Das et al. [36]	2016	USA	Sepsis	36.0	174	16 (9.19%)
Caldas et al. [9]	2019	Brazil	Mortality and NEC	36.5	4356	2339 (53.7%)
Ng’eny et al. [11]	2019	South Africa	Mortality and metabolic acidosis	36.0	799	453 (56.7%)
Yu et al. [5]	2020	China	Mortality, RDS, IVH, NEC, and sepsis	36.5	1247	1100 (88.2%)

**Fig. 2:**
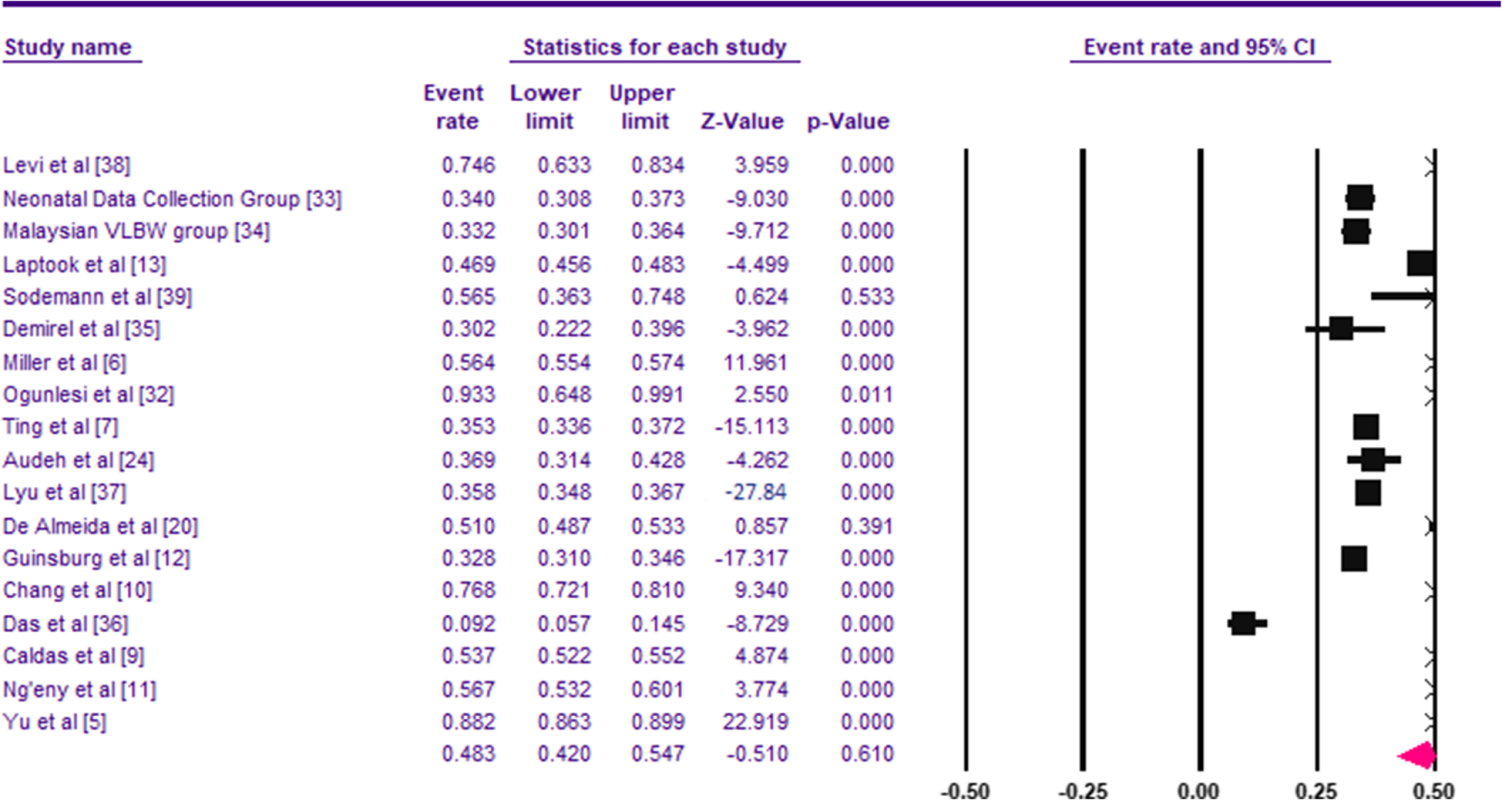
Pooled prevalence of HT among VLBW infants

**Fig. 3:**
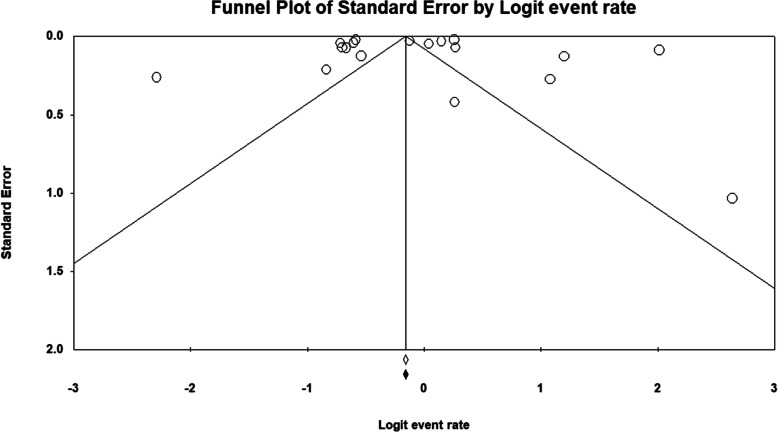
Funnel plot showing no evidence for publication bias among the studies included in this meta-analysis

Among the 40,136 VLBW infants included in the analysis, the pooled prevalence of HT among VLBW infants was 48.3% (95% CI, 42.0–54.7%) (Fig. 2). No evidence of publication bias was detected based on examination of the funnel plot and from the results of Begg’s test (*p* = 0.35) and Egger’s test (*p* = 0.37) (Fig. 3). The meta-regression showed that the prevalence of HT was not affected by the study year (*P* = .721).

### Risk of mortality among hypothermic VLBW infants

**Fig. 4:**
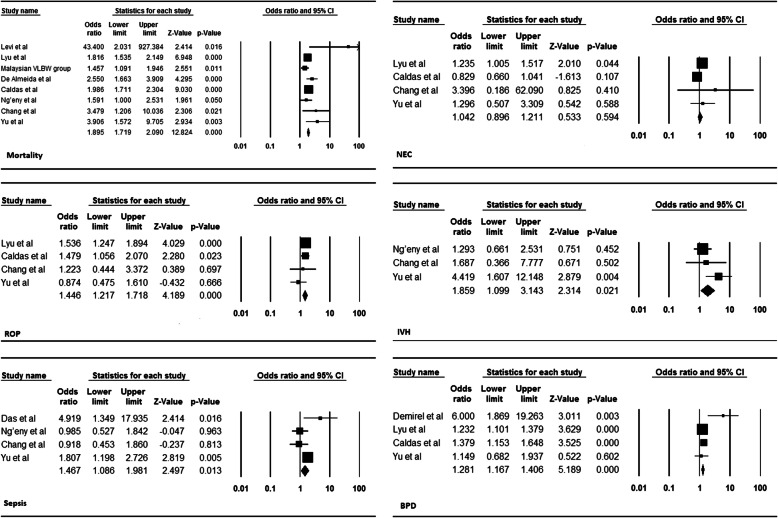
Pooled OR of Mortality NEC, IVH, BPD, Sepsis, and ROP among hypothermic VLBW infants

Eight studies had sufficient data to calculate the risk of death among hypothermic VLBW infants compared to the non-hypothermic VLBW population [5, 9–11, 20, 34, 37, 38]. The hypothermic VLBW infants have a higher risk of mortality with a pooled OR of 1.89 (95% CI, 1.72–2.09) (Fig. 4) (Table. 2). Duvall and Tweedie’s trim and fill method indicated three potential studies missing. However, the adjusted estimate was still significant and almost similar to the original findings after the virtual studies were appended, indicating that the result of this meta-analysis was steady (OR = 1.86, 95% CI, 1.69–2.05).

**Table 2:** Meta-analyses of the adverse outcomes associated with HT in VLBW infants

Adverse outcome	No. of studies	Pooled OR (95% CI)	***p*** value for OR
**Death**	**8**	1.89 (95% CI, 1.72–2.09)	*P* < .001
**NEC**	**4**	1.04 (95% CI, 0.89–1.21)	*P* = .594
**IVH**	**3**	1.86 (95% CI, 1.09–3.14)	*P* = .021
**BPD**	**4**	1.28 (95% CI, 1.16–1.40)	*P <* .001
**Sepsis**	**4**	1.45 (95% CI, 1.22–1.49)	*P <* .001
**ROP**	**4**	1.46 (95% CI, 1.08–1.98)	*P* = .013

### Risk of necrotizing enterocolitis (NEC) among hypothermic VLBW infants

Four studies had sufficient data to calculate the risk of NEC among hypothermic VLBW infants compared to the non-hypothermic VLBW population [5, 9, 10, 37]. There was no significant difference between the two groups and the pooled OR was 1.04 (95% CI, 0.89–1.21) (Fig. 4) (Table. 2). Duvall and Tweedie’s trim and fill method indicated one potential study missing. However, the adjusted estimate was still insignificant and almost similar to the original findings after the virtual study was appended, indicating that the result of this meta-analysis was steady (OR = 1.03, 95% CI, 0.76–1.42).

### Risk of intra-ventricular hemorrhage (IVH) among hypothermic VLBW infants

Three studies had sufficient data to calculate the risk of developing IVH among hypothermic VLBW infants compared to the non-hypothermic VLBW population [5, 10, 11]. The hypothermic VLBW infants have a higher risk of IVH with a pooled OR of 1.86 (95% CI, 1.09–3.14) (Fig. 4) (Table. 2).

### Risk of bronchopulmonary dysplasia (BPD) among hypothermic VLBW infants

Four studies with sufficient data to calculate the risk of BPD among hypothermic VLBW infants compared to the non-hypothermic VLBW population [5, 9, 35, 37]. The hypothermic VLBW infants have a higher risk of BPD with a pooled OR of 1.28 (95% CI, 1.16–1.40) (Fig. 4) (Table. 2). Duvall and Tweedie’s trim and fill method indicated one potential study missing and the adjusted estimate was (OR = 1.26, 95% CI, 0.95–1.69).

### Risk of neonatal sepsis among hypothermic VLBW infants

Four studies with sufficient data to calculate the risk of neonatal sepsis among hypothermic VLBW infants compared to the non-hypothermic VLBW population [5, 9, 10, 37]. The hypothermic VLBW infants have a higher risk of sepsis with a pooled OR of 1.47 (95% CI, 1.09–2.49) (Fig. 4) (Table. 2).

### Risk of retinopathy of prematurity (ROP) among hypothermic VLBW infants

Four studies with sufficient data to calculate the risk of ROP among hypothermic VLBW infants compared to the non-hypothermic VLBW population [5, 10, 11, 36]. The hypothermic VLBW infants have a higher risk of ROP with a pooled OR of 1.45 (95% CI, 1.28–1.72) (Fig. 4) (Table. 2).

## Discussion

In this review, we provided a comprehensive view of the prevalence of HT and its associated outcomes in VLBW infants. The prevalence of HT among VLBW infants was high and our result is close to a 2020-systematic review which reported that the prevalence of neonatal HT in East African countries was 57.2% [40]. Most of the studies included in our meta-analysis defined HT as axillary body temperature below 36.0 °C or 36.5 °C. There were two studies that used different cut-off points for the definition of HT; Audeh et al. found that 36.9% of the 271 VLBW infants in three Israeli centers had body temperature below 35.5 °C and Sodemann et al. in a cohort study conducted in Guinea-Bissau found that body temperature below 34.5 °C significantly increased the risk of death by approximately five times in the first 7 days of life [24, 39].

The adjusted multivariate analyses in the reviewed studies showed that HT was linked to increased risk of one or more adverse outcomes in VLBW infants, indicating that these findings were more than mere associations in the context of prematurity. Furthermore, Yu et al. showed that the correlation between HT and mortality was quadratic function equation and Lye et al. showed that the relationship between body temperature and adverse neonatal outcomes was U-shaped, in which the lowest rates of adverse outcomes were associated with the normal temperatures [5, 37].

These findings are explainable from the perspective of the pathophysiology of HT. Few studies provided potential explanations for the pathophysiological mechanisms by which neonatal HT lead to consequent mortality and adverse clinical outcomes [41]. It has been reported that HT affects virtually all systems causing wasting of the body’s stores of carbohydrate, protein, and fat due to the effect of the stress hormones such as cortisol and catecholamines, transient increased heart rate followed by bradycardia and serious electrocardiographic changes, decreased blood flow to the vital organs secondary to HT-mediated decrease in cardiac output, increased blood viscosity, and unpredictable fluctuations in serum electrolytes [41]. It has been shown that with prolongation of HT, the body’s oxygen consumption declines at a rate of 6% with each degree fall in body temperature with an inverse relationship between heat lost and serum glucose level [41].

The current review further confirmed the association of HT with VLBW neonatal deaths. The risk of mortality among hypothermic infants was reported as being highest within the first 7 days of life [11]. HT increased death rates by 39.5, 42, and 64% according to the findings of Guinsburg et al., Ting et al., and De Almeida et al., respectively [7, 12, 20]. Ogunlesi et al. showed that the case fatality rate (CFR) was significantly higher for hypothermic babies, especially in babies who weighed < 1 kg [32]. It is noteworthy that the incidence of mortality varied with varying degrees of HT [6]. A recent review showed that HT-associated mortality is related to the quality of neonatal care and it could be used as a quality indicator [42]. Simiyu et al. found that the mortality rate among low birth weight infants with HT was 84% [43]. In addition, a comparison of admission temperature between survivors and non-survivors showed a significant difference where the average body temperature was 35.4 °C and 34.8 °C respectively [44].

However, it is worth noting that attributing the poor neonatal outcome to HT might in some cases be tricky. In three groups of VLBW infants (750–999, 1000–1249, and 1250–1500 g) for instance as noted by Joelle et al., despite the significantly higher incidence of HT in the smallest infants compared to the (1250–1500 g) infants, HT was only significantly more common in the subgroup of infants who died in the larger groups, but not in the smallest infants group [45].

Our analysis concluded that there was an association between HT and IVH. Miller et al. found the association was with moderate HT specifically, whilst Yu et al. found the association with all types of HT [5, 6]. Guinsberg et al. found in their study that the IVH acquired by neonates due to HT was specifically of the IVH grade 3–4 [12].

Studies regarding NEC were controversial, and we did not detect an association between neonatal HT and NEC. Guinsburg et al. reported that NEC contributed synergistically to the development of unfavorable outcomes along with HT [12]. Caldas et al. described the significant protective association between admission HT and NEC like previous reports which showed that mild induced therapeutic HT may have some protective effect as rescue therapy for NEC in preterm infants [9].

Neonatal sepsis being linked to HT could be attributed to facts reported in the adult population undergoing surgery, where peri-operative HT was found to propagate post-operative infectious complications through temperature-mediated impaired immune function [13]. In a quality improvement project, the risk of nosocomial sepsis was significantly lowered in the period after the intervention to reduce neonatal HT [25]. However, a number of confounding factors have been established to contribute to the development of sepsis including the length of hospital stay and level of prematurity of the infant [36].

A limitation to be considered in this review is the paucity of publications on the associated outcomes with HT, which would be useful to reflect since several countries reported a high prevalence of neonatal HT, nonetheless still neglect the lack of thermal protection needed for newborn survival [2, 40, 41]. In such a situation, the magnitude of the burden would help emphasize the importance of interventional measures to be taken. Another limitation is the inclusion of studies published only in English, which could compromise representativeness. Lastly, data on the risk of several adverse outcomes were obtainable from few studies. There is a need for large prospective studies with appropriate controls to elaborate more on these issues.

## Conclusions

Neonatal HT rate is high in VLBW infants and it is a vital risk factor for mortality and morbidity among them. Healthcare providers need to be aware of the clinically important association between HT and adverse clinical outcomes to ensure effective management.

## Data Availability

The datasets used during the current study are available from the corresponding author on reasonable request.

## Abbreviations

VLBW: Very Low birth weight: HT: Hypothermia
NEC: Necrotizing enterocolitis
IVH: Intra-ventricular hemorrhage
ROP: Retinopathy of prematurity
BPD: Bronchopulmonary dysplasia
CFR: Case fatality rate
